# Apothecial Ancestry, Evolution, and Re-Evolution in *Thelebolales* (*Leotiomycetes, Fungi*)

**DOI:** 10.3390/biology11040583

**Published:** 2022-04-11

**Authors:** Luis Quijada, Neven Matočec, Ivana Kušan, Joey B. Tanney, Peter R. Johnston, Armin Mešić, Donald H. Pfister

**Affiliations:** 1 Department of Organismic and Evolutionary Biology, The Farlow Reference Library and Herbarium of Cryptogamic Botany, Harvard University, 22 Divinity Avenue, Cambridge, MA 02138, USA; dpfister@oeb.harvard.edu; 2 Laboratory for Biological Diversity, Ruđer Bošković Institute, Bijenička Cesta 54, HR-10000 Zagreb, Croatia; nmatocec@irb.hr (N.M.); amesic@irb.hr (A.M.); 3 Pacific Forestry Centre, Canadian Forest Service, Natural Resources Canada, 506 Burnside Road, Victoria, BC V8Z 1M5, Canada; joey.tanney@nrcan-rncan.gc.ca; 4 Manaaki Whenua Landcare Research, Private Bag 92170, Auckland 1072, New Zealand; johnstonp@landcareresearch.co.nz

**Keywords:** ancestry, apothecia, cleistothecia, *Holwaya*, *Holwayaceae*, *Patinella*, *Ramgea*, traits

## Abstract

**Simple Summary:**

*Leotiomycetes* is one of the most speciose classes of the phylum *Ascomycota* (*Fungi*). Its species are mainly apothecioid, paraphysate, and possess active ascospore discharge. *Thelebolales* are a distinctive order of the *Leotiomycetes* class whose members have mostly closed ascomata, evanescent asci, and thus passively dispersed ascospores. Within the order, a great diversity of peridia have evolved as adaptations to different dispersal strategies. The genus *Thelebolus* is an exceptional case of ascomatal evolution within the order. Its species are the most diverse in functional traits, encompassing species with closed ascomata and evanescent asci, and species with open ascomata, active ascospore discharge, and paraphyses. Open ascomata were previously suggested as the ancestral state in the genus, these ascomata depend on mammals and birds as dispersal agents. In our work, we used morphological and phylogenetic methods, as well as the reconstruction of ancestral traits for ascomatal type, asci dehiscence, the presence or absence of paraphyses, and ascospore features to explore evolution within *Thelebolales*. We demonstrate the apothecial ancestry in *Thelebolales* and propose a new hypothesis about the evolution of the open ascomata in *Thelebolus* involving a process of re-evolution where the active dispersal of ascospores appears independently twice within the order. A new family, *Holwayaceae*, is proposed within *Thelebolales*, comprising three genera: *Holwaya*, *Patinella*, and *Ramgea*.

**Abstract:**

Closed cleistothecia-like ascomata have repeatedly evolved in non-related perithecioid and apothecioid lineages of lichenized and non-lichenized *Ascomycota*. The evolution of a closed, darkly pigmented ascoma that protects asci and ascospores is conceived as either an adaptation to harsh environmental conditions or a specialized dispersal strategy. Species with closed ascomata have mostly lost sterile hymenial elements (paraphyses) and the capacity to actively discharge ascospores. The class *Leotiomycetes*, one of the most speciose classes of *Ascomycota*, is mainly apothecioid, paraphysate, and possesses active ascospore discharge. Lineages with closed ascomata, and their morphological variants, have evolved independently in several families, such as *Erysiphaceae*, *Myxotrichaceae*, *Rutstroemiaceae*, etc. *Thelebolales* is a distinctive order in the *Leotiomycetes* class. It has two widespread families (*Thelebolaceae*, *Pseudeurotiaceae*) with mostly closed ascomata, evanescent asci, and thus passively dispersed ascospores. Within the order, closed ascomata dominate and a great diversity of peridia have evolved as adaptations to different dispersal strategies. The type genus, *Thelebolus*, is an exceptional case of ascomatal evolution within the order. Its species are the most diverse in functional traits, encompassing species with closed ascomata and evanescent asci, and species with open ascomata, active ascospore discharge, and paraphyses. Open ascomata were previously suggested as the ancestral state in the genus, these ascomata depend on mammals and birds as dispersal agents. In this scheme, species with closed ascomata, a lack of paraphyses, and passive ascospore discharge exhibit derived traits that evolved in adaptation to cold ecosystems. Here, we used morphological and phylogenetic methods, as well as the reconstruction of ancestral traits for ascomatal type, asci dehiscence, the presence or absence of paraphyses, and ascospore features to explore evolution within *Thelebolales*. We demonstrate the apothecial ancestry in *Thelebolales* and propose a new hypothesis about the evolution of the open ascomata in *Thelebolus*, involving a process of re-evolution where the active dispersal of ascospores appears independently twice within the order. We propose a new family, *Holwayaceae*, within *Thelebolales*, that retains the phenotypic features exhibited by species of *Thelebolus*, i.e., pigmented capitate paraphyses and active asci discharge with an opening limitation ring.

## 1. Introduction

Fungi are among the most important organisms in the Earth’s ecosystems, but at the same time, they are one of the most poorly understood and studied groups. Little is known about their diversity; only 148,000 of the estimated 12 million species have been discovered [1,2]. Fungal forms can be simple and microscopic, such as the unicellular yeasts, or complex and multicellular, with intricate and extensive networks of hyphae [3,4,5,6,7]. *Ascomycota* is the largest and most diverse phylum of *Fungi* and they play key ecological roles as symbionts and saprobes in all terrestrial and aquatic ecosystems. The synapomorphy for the phylum is the specialized cell, a meiosporangium, called the ascus. Asci can be globose to sac-like to cylindrical, within which meiospores (ascospores) are produced. Unicellular species of the phylum have solitary asci (e.g., yeasts). Multicellular species either produce naked asci directly from hyphae, as in some *Saccharomycotina* and *Taphrinomycotina* (basal lineages of *Ascomycota*), or in multicellular structures, ascomata, that vary in shape, the mode of the presentation of the asci, and the development of associated structures (excipula and paraphyses) [6,8,9,10]. We can differentiate three main types of multicellular ascomata: open (apothecia), closed (chasmothecia, cleistothecia, gymnothecia, stereothecia), and partially closed (perithecia). Ascomatal types were used in the past to establish higher-level classification in *Ascomycota* [11]. Today, we know that closed ascoma have evolved independently in several apothecial lineages. Therefore, this ascomatal type is a homoplastic feature, driven in part by adaptation to environmental factors, and cannot be used at higher levels in phylogenetically-based classifications [9,11,12,13,14].

Morphological and functional variation in asci, ascospores, the presence of paraphyses, and variation in ascomatal construction are frequently correlated with ascoma type and dispersal strategies [13,15,16,17,18]. Asci have a variety of structural features including wall layering, the thickness of the apex, amyloidity, and dehiscence mechanisms (pore, slit, operculum, etc.). Asci with active ascospore discharge are often linked to open ascoma, where asci are arranged in a palisade manner forming a hymenium. At discharge, their rigid walls and turgor allow the asci to act like water cannons. Interascal space is occupied by paraphyses, suggested as important in contributing to the ejection of ascospores by contributing lateral pressure. For effective dispersal through the air, ascospores need to reach the zone of turbulent air and avoid the layer of still air that surrounds the ascoma [19,20,21], thus allowing them to disperse greater distances. Fritz et al. [22] correlated spore shape and size with ascus pore size. For successful discharge, the pressure within the ascus is proportional to the elasticity of the apical pore. This avoids the disorganized rupture of the ascus during discharge and maximizes launch distance without wasting energy [22]. On the other hand, species with evanescent asci do not actively discharge spores and usually have closed ascoma; the ascal walls are fragile and disintegrate, and the entire ascomata may become the dispersal unit, or spores are released passively by microbial breakdown or the rupture of the ascomatal wall [16,23,24,25,26]. Species with evanescent asci depend on the passive transport of the ascoma or spores, such as being blown off by wind, splashed off by rain or water currents, shaken off, or adhering to or being consumed by animals [16,18,19,27].

Ascospores are structures that function in fungal survival and/or dispersal. They vary in size, shape, wall ornamentation, color, contents, cell number, and the presence or absence of sheaths or appendages [19,27]. The morphological variation of ascospores may be informative about the ecology and dispersal abilities of a fungus [28]. Spore size and shape influence how far spores can be carried before deposition and have been correlated with their trophic status, such as saprobes versus mycorrhizae [18,19,29,30]. For example, large spores tend to be deposited closer (by impaction or sedimentation) to the place of origin, are less sensitive to desiccation and UV radiation because of the surface/volume relationship (i.e., narrow-long spores), and tend to be deposited above ground (i.e., allantoid spores) [16,18,19,29,31,32,33]. On the other hand, small air or water-borne spores have been correlated with long-distance dispersal. Depending on atmospheric factors, these spores can travel even between continents [29,30,34]. Wall thickness, hydrophobicity, and ornamentation are features that vary among species and are correlated with resistance to drying, animal dispersal (internal and external), reducing settling velocities, and trophic strategy [18,28,35,36]. Ascospore color also varies among species. Dark-colored spores vary from light brown to nearly black depending on the melanin content of the walls. Species with dark spores are dominant in certain ecosystems such as hot deserts [37,38]. Wall pigmentation has been correlated with a lower incidence of UV damage in experiments [28,37,38,39]. In general, melanized cells (hyphae, spores, and other propagules) are afforded protection against diverse environmental stresses, such as extremely low temperatures [40,41], hypersalinity [42], heavy metal toxicity [43], resistance to microbial lysis [44,45], and host defenses [46,47].

*Leotiomycetes* is one of the most diverse classes in the subphylum *Pezizomycotina*. *Leotiomycetes* species are widespread in terrestrial and aquatic ecosystems; many are plant pathogens or saprobes, but members of the class are also reported as endophytes, in mycorrhizas, and even with lichenized lifestyles [14,25,48,49,50,51,52,53]. Apothecioid ascomata and asci with active discharge by a pore have been suggested as ancestral features for *Pezizomycotina* as well for *Leotiomycetes*; therefore, closed ascoma and evanescent asci represent a repeatedly evolved, homoplastic morphology [13,16,26,54,55,56]. Cleistothecial-like ascomata appear in apothecial-dominated families of *Leotiomycetes* such as in *Rutstroemiaceae* (*Bicornispora*) and *Helotiaceae* (*Amylocarpus*). There are species with apothecia in cleistothecial-dominant orders, such as *Thelebolales*, whereas some families are comprised only of cleistothecial species, e.g., *Amorphothecaceae*, *Erysiphaceae*, *Myxotrichaceae*, *Pleuroascaceae*, and *Pseudeurotiaceae* [14,25,57].

*Thelebolales* is an exceptional case considering ascomatal evolution within *Leotiomycetes*. Currently, the phylogenetic placement of *Thelebolales* is clearly inside *Leotiomycetes* [14]. The order *Thelebolales* constitutes 11 genera and ca. 30 spp. [25,58,59]. Within *Thelebolaceae*, *Thelebolus* is the most speciose genus and the most diverse regarding functional traits. It encompasses species with closed ascomata that have asci without active discharge. Additionally, there are other genera with ascomata that expose asci late in their development, these resemble apothecia and have active ascospore discharge and paraphyses. Hoog et al. [60] provided the first molecular insights and hypothesized about the evolution of these *Thelebolus* species after species were found to be abundant in Antarctic lakes. Hoog et al. [60] postulated that open ascomata and active ascospore discharge in *Thelebolus* species (e.g., *T. stercoreus* and *T. microsporus*) are ancestral states in the genus, and suggested that lineages with closed ascoma and evanescent asci evolved as new lineages in Antarctica. Examples of the latter are *T. ellipsoideus* and *T. globosus*. These were suggested to have evolved in response to the harsh climate and loss of bird or mammal dispersal [60]. Their hypothesis correlates open ascoma with active discharge and agrees with the current knowledge about the ancestral traits of *Ascomycota*, but they did not consider re-evolution as a possible event inside *Thelebolales* [13,16,26,54,55,56]. The evolutions of traits should be studied on a larger scale to include several genera and involve both related taxa (ingroups) and outgroups. Given the expanded sampling and new sequences available since their publication, we believe Hoog et al.’s [60] hypothesis about the ancestral traits of *Thelebolus* should be revisited.

Taxon sampling in molecular phylogenetic studies is limited in *Leotiomycetes* [14,61,62]. This has hindered the comprehensive study of the systematics, ecology, and evolution in the class. Recent increases in the number and diversity of available DNA sequences for taxa of *Leotiomycetes* have improved the understanding of relationships among lineages in the class and, for the first time, have provided support for deep nodes. In relationship to the present study, Johnston et al. [14] found the genus *Holwaya*, previously placed in *Tympanidaceae*, to be sister to *Thelebolales*. *Holwaya* has a unique morphology: large, black, stipitate apothecia growing on coarse woody debris, and long, multiseptated ascospores that produce conidia in a more or less spiral pattern along the main spore axis. This combination of characters makes the genus remarkable and distinguishes it from its closest relatives. *Holwaya* comprises only two species, *H. mucida*, well-known worldwide from both its teleomorph and anamorph, and *H. byssogena*, only known from its anamorph. The asexual fungus most similar to these conidial states is *Neocrinula* (*Neocrinulaceae*), as noted by Crous et al. [63]. The second and third authors found a close phylogenetic relationship between *Holwaya mucida*, *Patinella hyalophaea*, and *Ramgea ozimecii* [64]*. Ramgea* is a coprophilous genus previously included in *Thelebolaceae* because of its morphological resemblance to *Thelebolus* [60,65].

Our aim is to investigate in depth the relationships of these open (apothecial) and closed (cleistothecial) ascomata lineages of *Thelebolales* using phenotypic characters, ecology, and molecular data to provide a reconstruction of ancestral traits. In their study, Fungi are among the most demanding large groups of organisms, and as aforementioned one of the most important groups of organisms for the key roles they play in the biosphere. We elaborate a model aimed at improving our understanding of evolutionary mechanisms in fungi. To accomplish this, we have used every available dataset presumably bearing evolutionary informative signals to provide an “integrative systematics” view of this group and to offer a more stable and functional taxonomy. In so doing, the evolutionary events that have led to high variability in morphology and functional adaptive traits in *Thelebolales* are explored.

## 2. Materials and Methods

### 2.1. Specimens Studied and Bibliographic Review

Fresh material collected by the authors and dried specimens were used for morphological and molecular studies. The new collections are deposited in the Farlow Herbarium (FH). Dried fungal specimens were obtained from the following herbaria/fungaria: C, CNF, CUP, FAMU and PRM (s. Index Herbariorum, http://sweetgum.nybg.org/science/ih (accessed on 1 February 2022)). A bibliographic review of genera and species in *Thelebolales* was conducted to gather information about the morphology, ecology, and distribution of the taxa. Records were found through HOLLIS (Harvard University’s online library catalog), Web of Knowledge, GBIF (Global Biodiversity Information Facility), and Google Scholar. In assembling the ecological data, we consulted a wide variety of sources, such as taxonomic articles, ecological reviews, checklists, papers concerned with molecular diversity and environmental DNA sampling, and the aforementioned trusted websites. Regarding terminology, we use “teleomorph” for ascigerous morph and “anamorph” for conidial morph, even though the terms “sexual” and “asexual morph” are widely used in newer literature, because in a number of anamorphic fungi, genetic recombination may normally occur via heterokaryosis without the formation of an ascigerous phase, while many conidial morphs function as spermatial producers, cf. Kirschner [66].

### 2.2. Morphological Studies

Morphological studies, together with cytological, cytochemical, and histochemical observations, were done following Baral [67], Quijada [68], and Kušan [69]. Macrophotographs of the ascomata on the substrate were taken of fresh samples in situ or after the rehydration of dried specimens. For microphotographs, we employed a Motic B1 compound light microscope with a USB Moticam 2500 camera, a Zeiss Axioskop 40 (Jena, Germany) with a Nikon D750 camera (Minato City, Tokyo) mounted on the microscope’s trinocular tube, and an Olympus BX 51 equipped with an Olympus DP 72 camera at magnifications up to 1000×. Sections for anatomical examination were made by hand using a double-edge safety razor blade or using a freezing microtome. The methods employed for the microtome are detailed by Karakehian et al. [70]. Fresh, living specimens were observed in tap water, Lugol’s solution ≈ 1% I_2_, 3% KI, aqueous (IKI), or 1% Congo Red (CR). Prior to microscopic analysis, sections made from dried collections were rehydrated in 3% potassium hydroxide (KOH), then treated with other reagents such as Congo Red (CR), Cotton Blue (CB), or Melzer’s reagent (MLZ). For ascomata, we characterized the macroscopic regions by differentiating the disc, margin, and receptacle. In sections, we studied the excipular structure of both the ectal and medullary excipula. In describing the ectal excipulum, the features of the base, lower and upper flank, and margin were characterized. Details of texture, cell shape, and pigmentation are provided. The hymenial elements (asci, ascospores, and paraphyses) were also described in detail and measured. The following abbreviations are also used in the text: * = living state; † = dead state; ↑ = spontaneously opened asci in taxa with forcible spore discharge; DIC = Differential Interference Contrast; OLR = opening limitation ring; TEM = Transmission Electron Microscopy. Color coding refers to Anonymous [71].

### 2.3. Phylogenetic Studies

Half of one dried apothecium from each *Holwaya* specimen was used for the molecular studies. DNA isolation, PCR reactions, and PCR amplification profiles followed Karakehian et al. [70]. Two datasets were used for two different analyses. The first dataset included sequences from the 15-gene dataset of Johnston et al. [14], together with *Lichinodiaceae* sequences from Prieto et al. [53], *Micraspidaceae* [72], the rDNA sequences available for *Neocrinulaceae* [63,73], and newly generated *ITS*, *LSU*, *TEF*, and *RPB2* sequences from *Holwaya mucida* specimens: LQH-102 (*ITS*: OM736084, *LSU*: OM736099, *TEF*: OM797039), C-F-91657 (*ITS*: OM736084, *LSU*: OM736100, *TEF*: OM797038), and CNF 2/8749 (*ITS*: OM282975, *LSU*: OM282978, *RPB2*: OM830434). *Neocrinulaceae* was added because of the morphological similarity between the anamorph of *Holwaya* and *Neocrinula* [63]. This dataset was used to explore the position of *Holwaya*, *Patinella*, and *Ramgea* with *Thelebolaceae* and *Pseudeurotiaceae* inside *Leotiomycetes*. The sequences available for each gene were aligned using the MAFFT v7.017 [74] plugin in Geneious 6.1.8 (https://www.geneious.com). The ends were manually trimmed, and introns were removed manually; all remaining data were then concatenated. Maximum likelihood (ML) analyses were run with IQ-TREE v.1.6.6 [75,76] using models selected by ModelFinder [77]; ultrafast bootstrap (BS) analysis with 1000 replicates estimated branch support in the ML tree [78]. *Xylaria hypoxylon* (*Xylariaceae*, *Xylariales*; AFTOL-ID 51, isolate OSC 100004, JGI genome Xylhyp) and *Neurospora crassa* (*Sordariaceae*, *Sordariales*; isolate OR74A, JGI genome Neucr2) were used as outgroups. Alignment, models for each partitioned gene, and sources of the sequence data for the taxa treated are provided as Supplementary Materials through the Manaaki Whenua–Landcare Research Datastore (https://doi.org/10.7931/X93K-H703). Illustrations were prepared in Adobe Illustrator CC (Adobe Systems, San Jose, CA). The second dataset included all taxa in Table 1 but with only one species representing *Holwaya*, *Patinella*, and *Ramgea*. *Neocrinula* was not included and some genera of *Leotiales* and related taxa were used as the outgroup (*Aotearoamyces*, *Claussenomyces*, *Microglossum*, *Myriodiscus*, *Thuemenidium*, *Tympanis*). This second dataset was used to perform the character state reconstruction using the morphological features explained in the following chapter.

### 2.4. Morphological Character Evolution within Thelebolales

**Figure 1:**
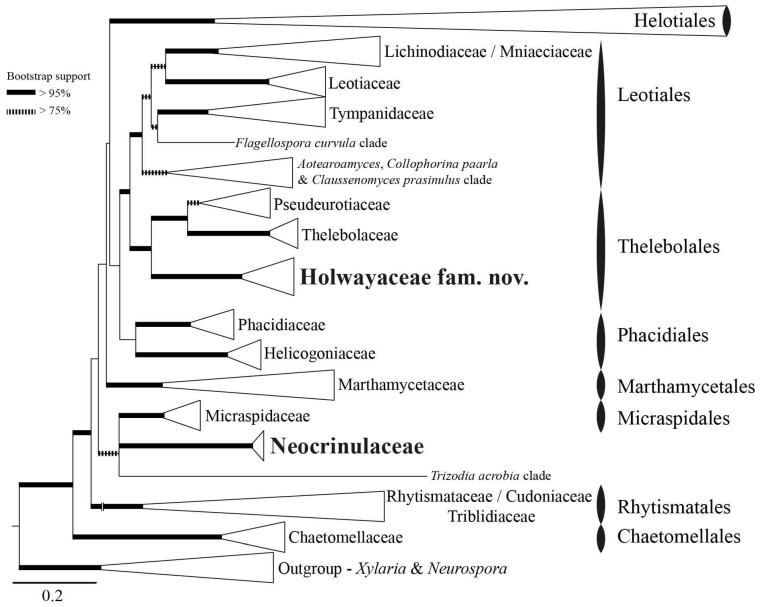
ML tree from concatenated DNA sequences. In bold we show the position of *Holwayaceae* fam. nov. and *Neocrinulaceae* inside *Leotiomycetes*. The collapsed clades represent the strongly supported family-level and order-level clades accepted by Johnston et al. [14]. Thick branches have bootstrap support (Bps) values > 95% and the dashed branches bootstrap support values 75–95%.

Six discrete phenotypic features (with character states in parentheses) were used to study the evolution inside the order *Thelebolales*: ascoma type (0 = open ascoma “apothecia”, 1 = closed ascoma “cleistothecia”, 2 = naked asci), ascospore discharge (0 = active discharge, 1 = passive discharge), paraphyses (0 = presence, 1 = absence), ascospore morphology (0 = cylindrical-fusiform-acicular, 1 = ellipsoid-ovoid-fusoid, 2 = globose-subglobose), ascospore color (0 = hyaline-yellowish, 1 = brown-dark), and ascospore ornamentation (0 = smooth, 1 = ornamented). The reconstruction of ancestral characters was performed using Mesquite v.3.6 [79]. The data matrix consisted of single species for each genus representing families in the order. We used 29 ingroup taxa and seven outgroup taxa that represent the closest related taxa according to our multigene phylogeny (Figure 1). *ITS* and *LSU* sequences were aligned with MAFFT v7.017 and trimmed with Gblocks v.091b [80]. The final alignment consisted of 1287 bp and is provided as Supplementary Materials through the Manaaki Whenua–Landcare Research Datastore (https://doi.org/10.7931/X93K-H703). We used the “Trace-characters-over-trees” command to calculate ancestral states at each node to plot the morphological traits onto 8000 input trees obtained from the MCMC analyses using MrBayes [81] through Geneious 6.1.7. (https://www.geneious.com) with the settings: 6,000,000 generations with sampling every 1500 generations, and a burn-in phase discarding the first 1000 sampled trees. The reconstruction of characters was performed with maximum parsimony and maximum likelihood using the Mk1 model.

## 3. Results

### 3.1. Phylogenetic Results

The overall topology of the ML phylogeny (Figure 1) is consistent with that presented by Johnston et al. [14], Quijada et al. [72], and Batista et al. [82]. The large *Helotiales* clade is collapsed because *Thelebolales* is outside of this order, positioned amongst the basal lineages within *Leotiomycetes* sister to *Leotiales* (Figure 1). Species of *Holwaya*, *Patinella*, and *Ramgea* form a strongly supported clade, formally named here as *Holwayaceae* fam. nov. As in the analysis of Johnston et al. [14], this clade has a strongly supported sister relationship to *Pseudeurotiaceae* and *Thelebolaceae* and is consequently accepted as a third family within the order *Thelebolales*. *Neocrinulaceae* is not related to *Holwayaceae* and appears isolated without a clear affiliation to any other family in our analysis (Figure 1).

### 3.2. Reconstruction of Ancestral States

**Figure 2:**
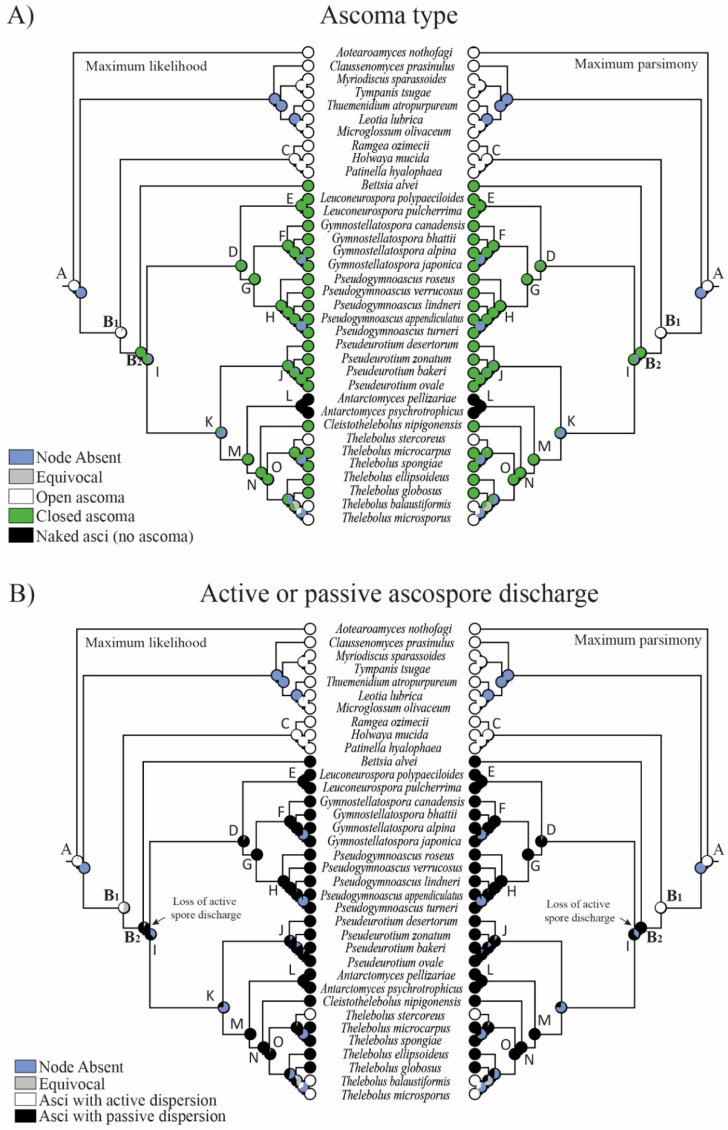
Evolution of (**A**) ascoma type and (**B**) active or passive ascospore discharge using parsimony and likelihood methods across 8000 BMCMC trees obtained from MrBayes. Pie charts at each node illustrate the likelihood (left) and parsimony (right) reconstruction and the proportion of the average received by each character state as the ancestral character of a given clade. Node absent indicates the proportion of nodes with posterior probabilities < 0.95 across trees. Equivocal indicates the same probability for the different features (not resolved).

**Figure 3:**
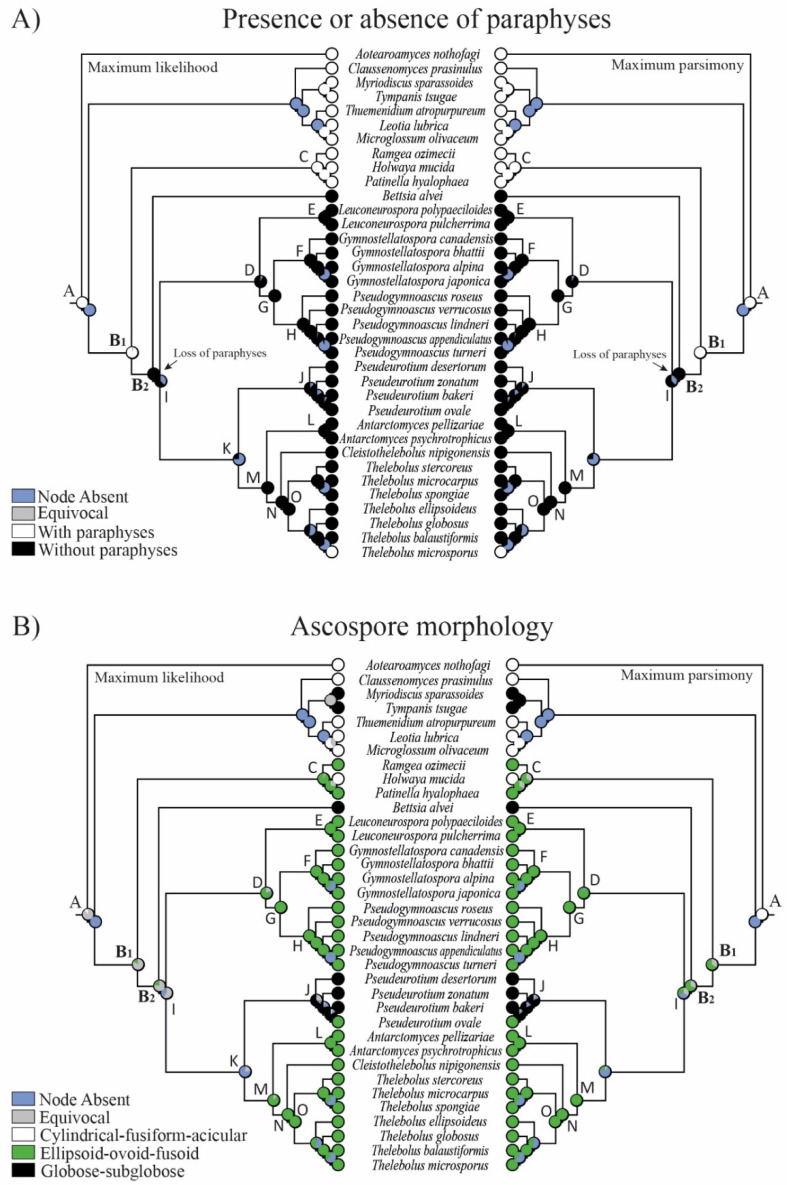
Evolution of (**A**) paraphyses and (**B**) ascospore morphology using parsimony and likelihood methods across 8000 BMCMC trees obtained from MrBayes. Pie charts at each node illustrate the likelihood (left) and parsimony (right) reconstruction and the proportion of the average received by each character state as the ancestral character of a given clade. Node absent indicates the proportion of nodes with posterior probabilities < 0.95 across trees. Equivocal indicates the same probability for the different features (not resolved).

**Figure 4:**
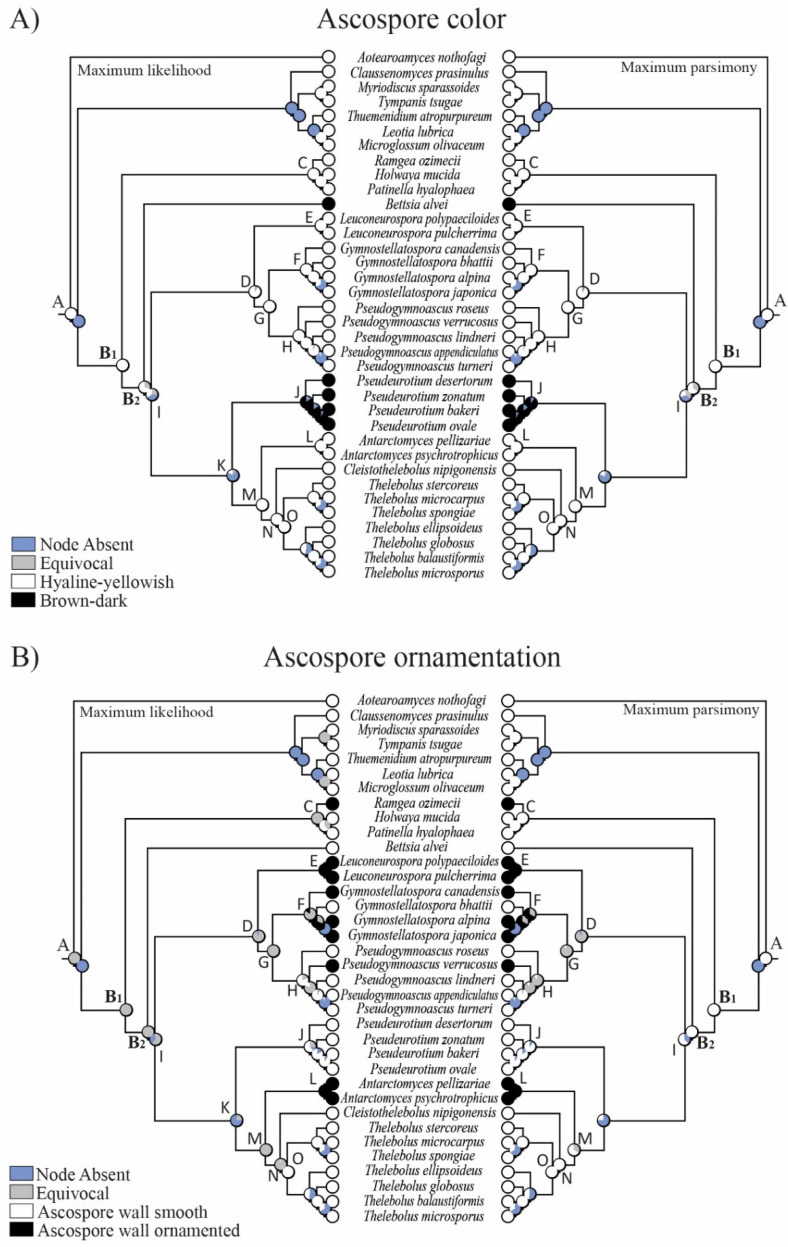
Evolution of (**A**) ascospore color and (**B**) ornamentation using parsimony and likelihood methods across 8000 BMCMC trees obtained from MrBayes. Pie charts at each node illustrate the likelihood (left) and parsimony (right) reconstruction and the proportion of the average received by each character state as the ancestral character of a given clade. Node absent indicates the proportion of nodes with posterior probabilities < 0.95 across trees. Equivocal indicates the same probability for the different features (not resolved).

**Figure 5:**
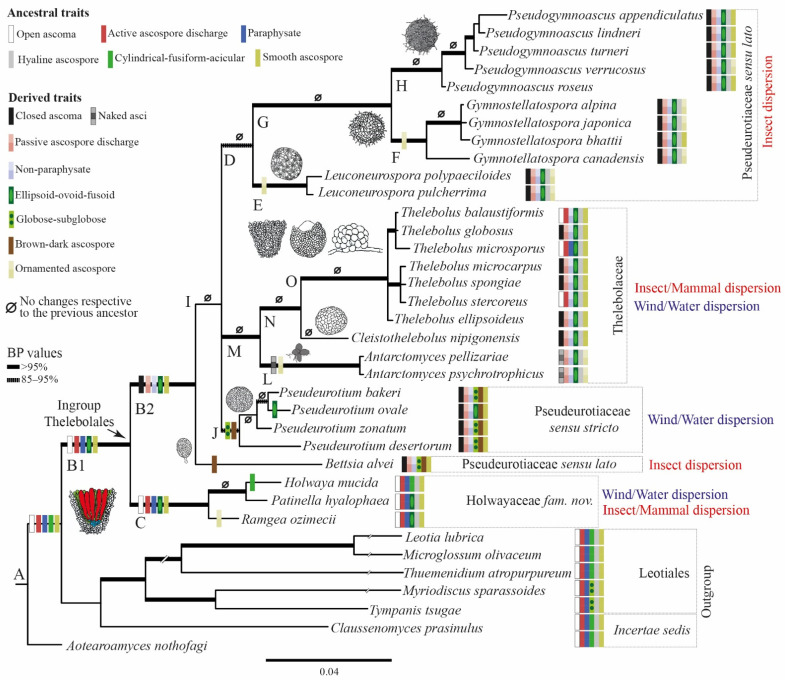
Hypothetical evolution of traits represented over the consensus Bayesian tree for *Thelebolales* based on the results of the reconstruction of ancestral states. Thick branches have bootstrap support (Bps) values > 95%. Ascoma morphology, as well as dispersal hypothesis, are represented for each clade.

One macro- and five micro-morphological characters were investigated in 16 clades (Figure 2, Figure 3, Figure 4 and Figure 5): A = common ancestor for outgroup and ingroup (*Leotiales*, *Thelebolales*); B1 = common ancestor for *Thelebolales*; B2 = common ancestor for the basal lineage *Bettsia*, *Pseudeurotiaceae* s.s. and s.l., and *Thelebolaceae*; C = common ancestor for *Holwayaceae* (*Holwaya*, *Patinella*, *Ramgea*); D = common ancestor for *Pseudeurotiaceae* s.l. (*Leuconeurospora*, *Gymnostellatospora*, *Pseudogymnoascus*); E = *Leuconeurospora* clade; F = *Gymnostellatospora* clade; G = common ancestor of *Gymnostellatospora* and *Pseudogymnoascus*; H = *Pseudogymnoascus* clade; I = common ancestor for *Pseudeurotiaceae* s.l. and s.s. and *Thelebolaceae*; J = *Pseudeurotium* clade s.s. (type genus in this clade); K = common ancestor for *Pseudeurotiaceae* s.s. and *Thelebolaceae*; L = *Antarctomyces* clade; M = common ancestor for *Thelebolaceae*; N = common ancestor for *Cleistothelebolus* and *Thelebolus*; and O = *Thelebolus* clade.

The reconstruction of ancestral characters showed complex macro- and micro-morphological patterns of evolution with the modification of ascomatal development and the gain or loss of different micro-morphological traits (Figure 2, Figure 3 and Figure 4). There are correlations between ascomatal development and active/passive spore discharge and/or presence/absence of paraphyses (Figure 2, Figure 3 and Figure 4). Open ascomata (apothecia) was inferred to be the ancestral state for *Thelebolales* (Figure 2 and Figure 5, clade B1), an order mostly comprising species with closed ascomata (cleistothecia). The common ancestor for *Pseudeurotiaceae* and *Thelebolaceae* was inferred as having a closed ascoma (Figure 2 and Figure 5, clades B2, I). *Bettsia alvei* produces the simplest closed ascoma, a unicellular cleistothecium (spore cyst), and is sister to all other lineages of *Pseudeurotiaceae* and *Thelebolaceae* (Figure 2 and Figure 5, B2). The major part of the descendant lineages of *Pseudeurotiaceae* and *Thelebolaceae* produce closed ascomata but with more complex multicellular covering layers than *Bettsia* (Figure 2 and Figure 5, clades D, E, G, H, J). The common ancestor for *Thelebolaceae* was inferred as having a closed ascoma (Figure 2 and Figure 5, clade M). *Antarctomyces* is reduced to naked asci produced directly on the hyphae (Figure 2 and Figure 5, clade L). The ancestor of *Cleistothelebolus* and *Thelebolus* was also inferred to produce closed ascomata (Figure 2 and Figure 5, clades N, O). Species with open ascoma are found throughout *Thelebolus*, e.g., *T. stercoreus*, *T. balaustiformis*, and *T. microsporus* (Figure 2 and Figure 5, clade O). The parsimony reconstruction indicates that the ancestor of *Thelebolales* had active ascospore discharge (Figure 2 and Figure 5, clade B1). The maximum likelihood reconstructions indicate the same, but 51% of the reconstructions are ambiguous (equivocal). Asci with active discharge appear only in *Holwayaceae* and some species of *Thelebolus* (Figure 2 and Figure 5, clades C, O). The ancestor and all the other clades of *Thelebolales* were inferred with non-active ascospore discharge (Figure 2 and Figure 5, clades B2, D, E, F, G, H, I, J, K, L, M, N, O). The ancestor of *Thelebolales* was inferred as paraphysate (Figure 3 and Figure 5, clade B1). The ancestor of *Holwayaceae* was inferred as paraphysate, but *Thelebolaceae* and *Pseudeurotiaceeae* were inferred as non-paraphysate (Figure 3 and Figure 5, clade B2). The presence of paraphyses in *Thelebolales* is linked to species with active ascospore discharge and open ascomata in both *Holwayaceae* and *Thelebolaceae*. In the *Thelebolus* lineage, only *T. microsporus* has paraphyses (Figure 3 and Figure 5, clades C, O). Maximum likelihood methods for ascospore morphology reconstruction were mostly equivocal for the ancestor of *Thelebolales*, but the parsimony method shows that 66% of the reconstructions are ellipsoid-ovoid-fusoid (Figure 3, clade B1). The internal nodes for *Pseudeurotiaceae* s.l. (Figure 3 and Figure 5, clade D) and *Thelebolaceae* (Figure 3 and Figure 5, clade M) keep the same morphology but the ancestor of *Pseudeurotiaceae* s.s. is reconstructed with globose-subglobose spores (Figure 3 and Figure 5, clade J). Hyaline-yellowish spore color was inferred as the ancestor of *Thelebolales*; only *Bettsia* and the ancestor of *Pseudeurotium* have brown-dark ascospores. Dark color and a globose-subglobose ascospore shape are known only in *Bettsia* and *Pseudeurotium* (Figure 4 and Figure 5, clades J, E). The evolution of ornamented spores appeared several times in independent lineages (Figure 4 and Figure 5): clade C “*Ramgea*”, clade E “*Leuconeurospora*”, clade F “*Gymnostellatospora*”, Clade H “*Pseudogymnoascus verrucosus*”, and clade L “*Antarctomyces*”. The common ancestor of *Holwayaceae*, *Pseudeurotiaceae*, and *Thelebolaceae* were inferred as smooth ascospores in the maximum parsimony reconstruction (Figure 4 and Figure 5, clades B, I, K, M, N), those nodes in the maximum likelihood analysis are mostly equivocal.

### 3.3. Taxonomy

*Holwayaceae* Quijada, Matočec, and I. Kušan, fam. nov.

**Figure 6:**
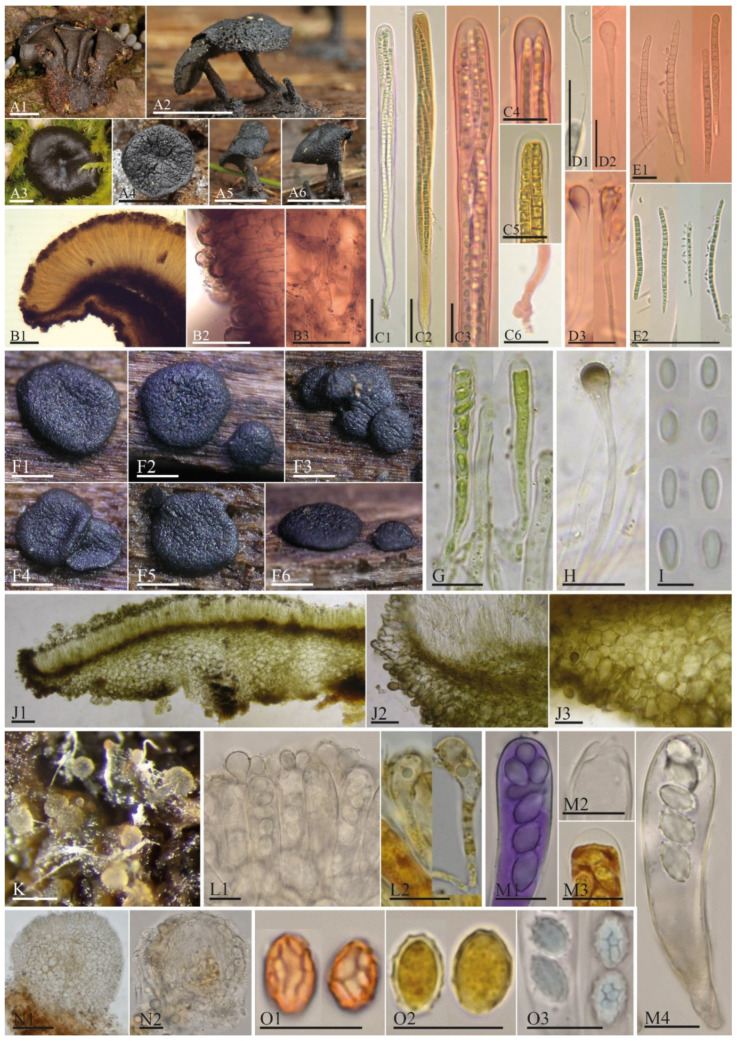
Phenotypic features of *Holwayaceae*: *Holwaya* (**A1**–**6**,**B1**–**3**,**C1**–**6**,**D1**–**3**,**E1**–**2**), *Patinella* (**F1**–**6**,**G**,**H**,I,J**1**–**3**) [84], and *Ramgea* (**K**,**L1**–**2**,**M1**–**4**,**N1**–**2**,**O1**–**3**) [64]. Comparative morphological features among genera in the family: A, F, and K. Macromorphology of the apothecia: B, J, and N. Excipulum in transverse section: C, G, and M. Asci and details of ascus apex: D, H, and L. Paraphyses: E, I, and O. Ascospores. A1, A3 from CNF 2/8749; A2, A4–6 from L.Q.H.-102; B1–3, C3–4, D2–E2 from CUP-60122; C1–2, C6, D1 from CUP-A-019509; C5 from CUP-D-02006. A1, A3 phot. N. Matočec, A2, A4–6 phot. J. Warfel, B1–E2 phot. Luis Quijada. Scale bars: A1–6 = 5 mm; F1–6, K = 0.5 mm; B1, N1 = 100 µm; B3, C1–2, D1, E2, J1 = 50 µm; J2 = 20 µm; B2, C3–6, D2–3, E1, G, H, J3, L1–2, M1–4, N2, O1–3 = 10 µm; I = 5 µm.

Mycobank number: MB 842621, Figure 6.

Type genus: *Holwaya* Sacc., Syll. fung. (Abellini) 8: 646 (1889)

Etymology: named after the type genus *Holwaya*.

Other genera included: *Patinella* Sacc., *Ramgea* Brumm.

Position in classification: Holwayaceae, Thelebolales, Leotiomycetes, Pezizomycotina, Ascomycota.

Diagnosis: Phylogenetically isolated within *Thelebolales*. Ascomata open; outer layer of the excipulum with globose to angular cells; capitate paraphyses with intra- or extracellular pigments and asci with forcible spore discharge augmented by an opening limitation ring-type apical apparatus; ascospores hyaline, smooth, or ornamented; either aseptate and ellipsoid-ovoid-fusoid, not producing conidia, or with more than ten septa, acicular-cylindrical-fusiform-acicular, and producing conidia. *Ramgea* differs from apothecioid members of *Thelebolaceae* by ornamented ascospores and paraphyses with capitate tips bearing granular, firmly cemented pigment exudates. *Patinella* and *Holwaya* differ from *Tympanis* and *Durandiella* (*Tympanidaceae*) and *Aotearoamyces* and *Bulgaria* (*Phacidiaceae*) in excipular composition of mostly globose-angular cells in combination with non-amyloid asci and ascospores.

Description: Ascoma apothecioid, paragymnohymenial or eugymnohymenial, superficial or erumpent, scattered to gregarious, solitary or sharing a common stroma-like base; pulvinate-turbinate to discoid or cup-shaped, pale yellowish to black, hymenium margin and receptacle concolorous, margin indistinct, thin and smooth, disc ± circular when fresh, sometimes slightly irregular by mutual pressure; sessile to short- to rather long-stipitate, stipe tapering downward; surfaces smooth or slightly rugulose due to the protrusion of paraphyses tips in the hymenium or protruding cells in the ectal excipulum. Asci cylindric-clavate, 4–8-spored, (1–)2–4-seriate; apex dome-shaped with a subapical OLR (best visible by the DIC of *ripe unopened and in ↑asci) with its thinner, lateral wall staining in CR but subapical ring and walls at apex unstained, slightly subconical and with moderately to strongly swollen wall in dead state, apex wall also partly laterally thickened; wall IKI and MLZ negative; base narrowed to a short to medium-long stalk and arising from croziers. Ascospores ellipsoid-ovoid-fusoid to cylindrical-fusiform-acicular, hyaline, straight or slightly to moderately curved, smooth to ornamented with coarse ridges or crests that can form a reticulum (KOH stable), eguttulate or multiguttulate (tiny oil drops); aseptate to (10–)16–20 transversal septa when septate and overmature with a small lateral wart-like to rod-shaped protuberance at each cell, on which one or two small conidia are formed. Paraphyses filiform, apically moderately to strongly clavate-capitate, exceeding living mature asci, sparsely branched at lower cells, ±equidistantly and sparingly septate; apical cells hyaline or partly olive-brown, pigmented with or without refractive globules, wall surface smooth to finely warted; cells apically free or agglutinated and embedded in a brownish gelatinous matrix and exudate. Subhymenium composed of compact ascogenous cells. Medullary excipulum very reduced and sometimes undifferentiated from ectal excipulum; if differentiated, then composed of dense *textura intricata*, slightly gelatinized, of thin-walled cells with a ±vertical orientation to the subhymenium. Ectal excipulum of ±*textura globosa*–*angularis* to *t*. *prismatica*, cells hyaline to olive-brown; cortical layer with groups of angular to clavate cells slightly protruding, thick-walled, covered by a dark, brownish exudate. Anchoring hyphae at base present or not. Anamorph: Only known for *Holwaya*. For morphological details see Seifert [83].

Specimens examined: *Holwaya mucida*—CANADA: State and province not recorded, on the bark of *Tilia* sp., 3 October 1896, *J. Macoun* (CUP-D-02006).—CROATIA: Primorje - Gorski kotar County, Nadvučnik village, near town Vrbovsko, the vegetation of young *Carpinus betulus* with *Pinus nigra* and *Picea abies*, on cut, semi-decorticated lying trunks of *Tilia* sp. covered with moss, 6 November 2010, *R. Kranjčev, N. Matočec and I. Kušan* (CNF 2/8749).—CZECH REPUBLIC: South Bohemian Region, NE of Hluboká nad Vltavou, Libochovka Nature Reserve, a forest of *Fagus sylvatica* with admixed *Picea abies* and *Tilia* sp., on a fallen trunk of *Tilia* ap. covered with mosses, 16 October 2016, *J. Holec* (PRM 944637).—DENMARK: Sjælland Region, Ringsted Municipality, Allindemagle Skov, forest with *Tilia* sp., on *Tilia* sp., 20 September 2011, *M. Vestergaard* (det. *Thomas Læssøe*) (C-F-91657)—SLOVENIA: Gorizia Region, Idrija, 370 m S-SE from the Kres peak (521 m), urban alley of *Tilia* sp. trees, on a rotten lying trunk of *Tilia* sp., 23 November 2001, *A. Piltaver and N. Matočec* (CNF 2/5423).—SWEDEN: Västmanland County, Västerås-Barkarö parish, Flaten island near Ridön island, on a fallen trunk of *Tilia cordata*, 20 October 1975, *S. Ryman* (PRM 869874).—USA: New Hampshire State, Carroll County, Chocorua, on *Acer* sp., September 1910, W.G. Farlow (PRM 685875, as *H. gigantea*), *idem* (PRM 685876, as *H. gigantea*); New York State, Slaterville Springs, Lloyd-Cornell Preserve, on a rotten log of *Tilia* sp., 25 September 1954, *R.P.Korf* (PRM 919564); *idem*, Coy Glen, Ithaca, on unidentified wood, 2 October 1982, *T. Capiello and N. Shishkoff* (CUP-60122); *idem*, Tompkins, Enfield, on unidentified wood, 14 October 1905, *G. Atkinson* (CUP-A-019509); Massachusetts, Concord, Estabrook Woods, on a fallen trunk of *Populus* sp., 3 Feburary 2019, *Luis Quijada* (L.Q.H.-102 in FH). *Ramgea ozimecii*—CROATIA: Lika-Senj County, near Perušić, about 2.6 km E-SE of Gornji Kosinj, pit Čardačina jama, in the dark zone of the karstic pit, on bat dropping, 30 September 2016, *R. Ozimec* (CNF 2/9997, holotype). *Patinella hyalophaea*—BOSNIA AND HERZEGOVINA: Sutjeska National Park, riverine canyon bottom, wet dicot woody remnant, 28 June 2015, *N. Omerović* (FAMU-1390, N.O.280615-03); Sarajevo County, Bijambare Protected Landscape, Brodić stream, periodical karstic watercourse in alti-montane conifer forest, *Picea abies* wet remnant, 4 August 2014, *N. Omerović* (no voucher).

## 4. Discussion

### 4.1. Diversity and Systematics of Holwayaceae

In recent classifications, *Thelebolales* has been placed in *Leotiomycetes* with two families, *Pseudeurotiaceae* and *Thelebolaceae*, with 8 and 12 genera, respectively, and three genera *incertae sedis* [25,59,85,86]. Here, we expand the concept of the order by adding a third family, *Holwayaceae*, constituting three genera (*Holwaya*, *Patinella*, *Ramgea*) previously placed in *Tympanidaceae*, *Thelebolaceae*, or *incertae sedis* in *Helotiales* or *Leotiomycetes* (*op cit.*). *Holwayaceae* is the basal lineage of the order (Figure 1 and Figure 5). *Holwaya* and *Ramgea* have only two species each, but *Patinella* has ca. 25 spp. [25,85]. There are no available sequences for *H. byssogena* (Berk. & Broome) Seifert or *R. annulispora* Brumm. Our taxonomic studies and bibliographic revision indicate that these two species are properly placed in these genera on morphological grounds. Even though the type species of *Patinella* is related to *H. mucida* and *R. ozimecii*, our unpublished studies of other species of *Patinella* indicate that the genus is highly polyphyletic, including species that should be placed in various families of *Leotiomycetes* or *Lecanoromycetes* (Quijada et al., unpubl. data). For this reason, our concept of *Holwayaceae* is circumscribed to currently include only one species of *Patinella*, the type species *P. hyalophaea* (Figure 6), but all accepted species of *Holwaya* and *Ramgea*.

The anamorphs of *Holwaya* and *Neocrinula* have similar morphology but the phylogenetic results in Crous et al. [63] did not suggest a close relationship, which was confirmed in other published phylogenies [14,86] as well as in our results (Figure 1). As Johnston et al. [14] stated, it is possible to consider a broader concept of *Leotiales* to include both apothecial lineages (*Holwayaceae*, *Lichinodiaceae*, *Mniaeciaceae*, *Tympanidaceae*) and those mostly reduced to cleistothecial ascomata (*Pseudeurotiaceae*, *Thelebolaceae*) (Figure 1). We prefer to keep two separate orders, given the similarities found among *Holwayaceae*, *Thelebolaceae*, and *Pseudeurotiaceae* (Figure 6), and to avoid changes in the unstable classification of the class given the lack of taxon sampling [14,85]. Finally, our results for *Pseudeurotiaceae* suggest polyphyly of the family, although we are only using one specimen per taxa and only teleomorphs (Figure 2 and Figure 5). Other studies that used several specimens with anamorphs and teleomorphs found monophyly of the family, but it did not include *Bettsia* [86]. For that reason, we here prefer to keep *Pseudeurotiaceae* and use sensu lato (s.l.) and sensu stricto (s.s.) for the clades.

### 4.2. Ecology and Distribution in Holwayaceae

*Holwayaceae* is so far comprised of saprobes on wood or dung [64,87,88,89,90]. *Holwaya mucida* is well known in the northern hemisphere (Palearctic, Nearctic), with most reports of it on fallen trunks of *Tilia*, and on other hardwood genera such as *Acer*, *Castanea*, *Fagus*, *Fraxinus*, *Magnolia*, *Quercus*, and *Ulmus*. The species is found in boreal forests or taiga and temperate broadleaf and mixed forests [87,88,91]. This species prefers old-growth forests, high atmospheric humidity, and anamorphs are more frequently found than teleomorphs [89]. The other species known only from the anamorph, *H. byssogena*, is found in tropical and subtropical dry or moist broadleaf forests (Neotropic realm) on unidentified wood, *Bactris*, and *Psidium* [83,91]. Both species of *Holwaya* are considered saprobes on wood [87,88,89], but little is known in detail about the ecology.

There are very few reports of *Patinella hyalophaea*. The discovery of *P. hyalophaea* in New Brunswick, Canada possibly represented its second collection since the original description of it from Italy in 1875 [84]. These authors observed similar climate and vegetation conditions of their collection site (coastal eastern Canada) and the type locality in montane northeastern Italy, including *Fagus* as the dominant forest tree species. Baral and Carter [84] also suggested an association with semi-aquatic habitats subjected to occasional flooding, which is in line given the recent report of *P. hyalophaea* from a torrent watercourse in a bog complex of an altimontane karst polje in the Dinaric Alps [92]. *Patinella hyalophaea* was isolated from historic wood in Deception Island, Antarctica [93], and most recently from buried wooden artifacts at five sites in Western Greenland and lacustrine sediment cores from a lake in King George Island, Antarctica [94,95]. *Ramgea annulispora* was described from pheasant (*Phasianus colchicus*) dung in the Netherlands [90], and *R. ozimecii* was described from bat droppings in Croatia [64]. A significant point in the evolution of *Holwayaceae* occurred between the basal *Ramgea* lineage and *Patinella-Holwaya* clade (Figure 5), when nutrition strategy might have switched from fimicolous saprotrophy to lignicolous saprotrophy. The evolution and nutritional switch in *Holwayaceae* are accompanied by several phenotypic changes (Figure 6) discussed below.

### 4.3. Evolution in Holwayaceae

**Figure 7:**
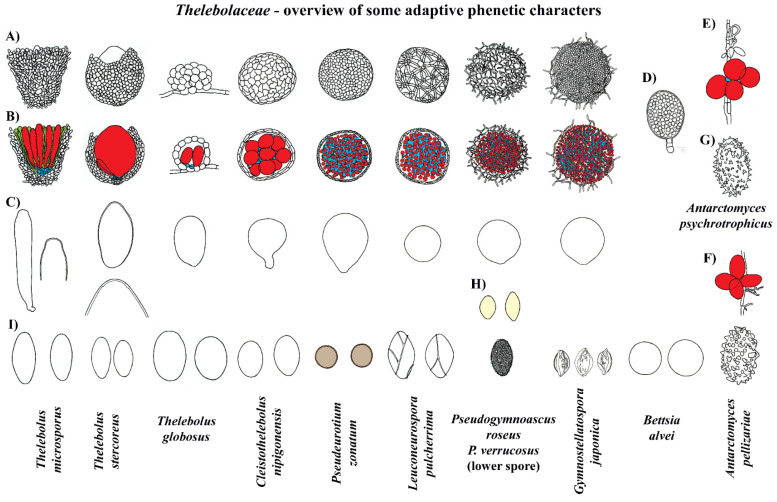
*Thelebolaceae*-an overview of some adaptive phenotypic characters. (**A**) Ascomata-side view. (**B**) Ascomata in section. (**C**) Asci (in *Thelebolus microsporus* and *T. stercoreus*, also enlarged view of ascus tips). (**D**) Cyst-like ascoma with ascospores inside. (**E**,**F**) Naked asci. (**G**,**H**,**I**) Ascospores. Color code: Red = asci; blue = ascogenous cells; green = hamathecium. Compiled drawings are not presented to scale in order to fit the single plate. Del. N. Matočec.

Our analyses indicate that the ancestral state of the group is apothecial-paraphysate with forcible spore discharge, as seen in the monophyletic family *Holwayaceae* (Figure 2, Figure 3, Figure 4 and Figure 5 clade C). The closed, non-paraphysate ascoma of *Thelebolaceae* and *Pseudeurotiaceae*, with the passive release of spores, is a derived condition (Figure 2, Figure 3, Figure 4 and Figure 5, clade B2). *Holwaya*, *Patinella*, and *Ramgea* share several traits: (1) open ascoma, (2) outer layer of the excipulum with globose/angular cells, (3) capitate paraphyses with intra- or extracellular pigments, and (4) asci with forcible spore discharge augmented by an OLR-type apical apparatus (Figure 2, Figure 3, Figure 4, Figure 5, Figure 6 and Figure 7). Here, we show that nutritional switches and habitat preferences in *Holwayaceae* are accompanied by several phenotypic modifications.

Fruitbody size is variable in the *Holwayaceae*. Changes in fruiting body size have been correlated with lifestyles in fungi [96]. Large fruiting bodies typically live longer, are more resistant to desiccation due to the lower surface-to-volume ratio, and increase the number of asci and ascospores per fruiting body, thus enabling a much higher production of propagules [97,98]. In *Holwayaceae*, larger ascomata occur in the woody saprobes (*Holwaya*, *Patinella*) compared to the dung saprobes (*Ramgea* spp.). Dung saprobes, such as *Ramgea*, must develop more rapidly to maturity due to the transient nature of their substrate and the spatial competition for limited resources [99]. The larger apothecia—up to 15 mm in diameter [87]—of *Holwaya mucida* are a striking deviation from the ascomata of *Ramgea* (0.04–0.36 mm diameter) [64,90]. *Patinella hyalophaea* produces apothecia that are intermediate in size (up to 1.2 mm in diameter) [84]. The production of relatively large, energy-demanding ascomata in *Holwaya* and *Patinella* is probably permitted by their ability to colonize wood, a substrate that is nutritionally, temporally, and spatially less restrictive compared to the ephemeral animal dung where the constrained colonies produced by *Ramgea* can develop only small fruiting bodies [99,100]. Remarkable differences in the ascomatal morphology of closely related taxa with contrasting trophic strategies can be seen in other *Leotiomycetes* taxa [61,101]. For example, *Bulgaria inquinans* (*Phacidiaceae*, *Phacidiales*) is a lignicolous saprotroph that produces exceptionally large and morphologically divergent ascomata compared to the more reduced ascomata of the endophytic and parasitic species typical of this family [102]. Another example is found in *Leotiales*, the sister order to *Thelebolales* (Figure 1), where small fruiting bodies are dominant in pathogens such as *Tympanidaceae* and *Mniaeciaceae*, but large fruiting bodies evolved in ectomycorrhizal lineages such as *Leotia* [14].

**Figure 8:**
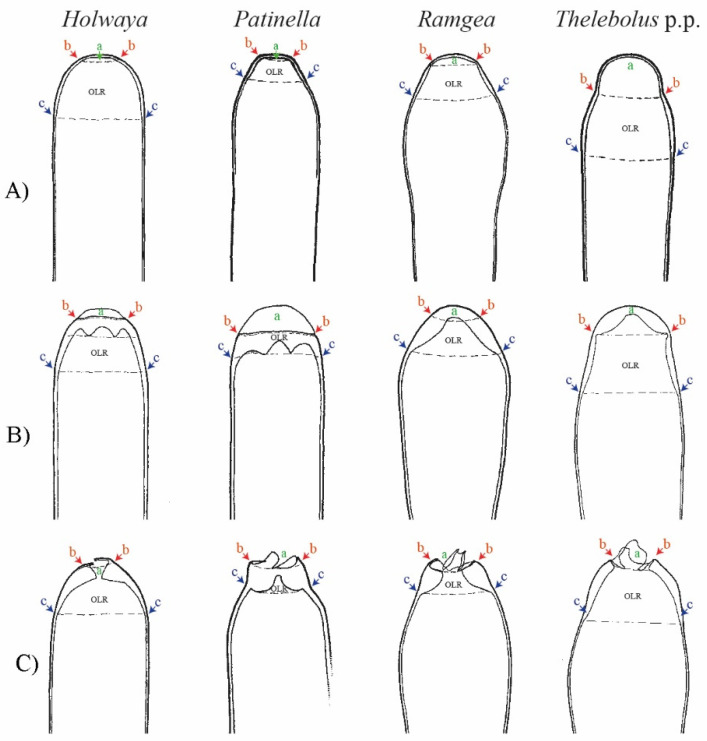
Asci apices with OLR-type dehiscence in *Thelebolales* species with forcible discharge asci: (**A**) mature ascus (ascal wall compressed by ascoplasm turgor); (**B**) mature ascus (ascal wall relaxed due to loss of turgor); (**C**) ↑ mature ascus displaying irregular (asymmetric) tear above OLR after ascospore discharge; a—opening apical zone above OLR; b—upper OLR line; c—lower OLR line. Compiled drawings are not presented to scale in order to fit the single plate. Del. N. Matočec.

The ancestor of *Holwayaceae* was reconstructed with active discharge asci (OLR) and hyaline ellipsoid-ovoid-fusoid ascospores (Figure 2, Figure 3, Figure 4 and Figure 5). Unlike the majority of *Leotiomycetes*, species of *Holwayaceae* do not possess a complex and stable structured plug-like apical pore, like in *Helotiales* [25,101], but rather a subapical wall reinforcement, like a ring (OLR) [65]. Its function is to limit tearing below the ring level, allowing opening above an irregular tear (Figure 8). *Patinella* and *Ramgea* retained the ancestral traits of the family. Their ascospores have the same shape (Figure 2 and Figure 5) and their apical opening is similar to, although wider than, *Holwaya* (Figure 8).

*Ramgea* is the only genus in the family with ornamented ascospores (Figure 6), a feature that is present in many animal-associated fungi [15,18,103], and discussed later here under the evolution of *Thelebolales*. *Holwaya* is the most divergent lineage inside the family and has developed several apomorphies in adaptation to its ecology. The OLR is positioned closer to the apex, producing a narrower opening. This correlates with the ascospores shape (narrow-long) (Figure 6) that has evolved to be less sensitive to desiccation (high surface/volume relationship), travel further in calm air, deposit above the ground by impaction [28], and resist wash-out by precipitation [18]. *Ramgea* and *Patinella* have ellipsoid, smaller ascospores that presumably can travel further than *Holwaya* [19,29,30]. However, *Holwaya* has a unique apomorphy: each cell of the ascospore produces up to several secondary spores (Figure 6), very small conidia (Figure 6, E1, E2. 3 × 0.5 µm). These might be specialized to penetrate deeper into the host substrate (e.g., bark furrows), to enable a further increase in propagule productivity per fruiting body and secure better long-distance dispersal [28,29,30,34], or to serve as spermatia. The conidia, if produced on ascospores that have landed on an unsuitable substrate, may facilitate subsequent dispersal to possibly more appropriate hosts.

Changes in pigmentation are also observed in *Holwayaceae* (Figure 6). Dark pigments might have evolved to protect against UV radiation, temperature fluctuation, desiccation, digestion by hydrolytic enzymes, or to absorb heat and warm ascomata quickly during a brief winter thaw [26,104,105]. The ascomata of the basal lineage *Ramgea* are hyaline or light-colored (Figure 6) [64,90], as are the fimicolous species in *Thelebolus* [60]. *Holwaya* and *Patinella* are completely black (Figure 6).

The most diagnostic synapomorphy of the *Holwayaceae* is found in the paraphyses, where protective KOH-inert pigmentation is present (Figure 6). More subtly defined apomorphies in the pigment location are confined to each genus either inside or outside the cell walls. In *Ramgea*, a light-yellowish paraphysal pigment is distributed within the wall and inside the cells, but a dark pigment is found only on the wall in *Patinella* and only outside the cell walls in *Holwaya* (Figure 6). In addition, paraphyses in *Holwayaceae* are abruptly enlarged at their tips. Brummelen [90] and Baral et al. [26] proposed that this shape could help to preserve the hydrated intercellular system of the hymenium and protect immature asci during their development. The latter authors also suggested that rough exudate surfaces over the paraphyses might assist condensation from atmospheric humidity (*op. cit.*). The large ascomata of *Holwaya* can survive under snow and reproduce during the autumn-winter thaw in the northern hemisphere [87] (Quijada pers. obs.). Its black tissues and paraphysal apomorphies effectively resist the desiccation of the hymenial layer, making the organism more capable of colonizing and reproducing in drier habitats [89] such as the surface of fallen trunks exposed to sunlight. *Patinella hyalophaea* dwells in more humid or even flooded habitats and does not exhibit, or consequently require, such an efficient paraphysal protective layer. On the other hand, the ascomata of *Ramgea*, with lighter pigmentation, are ephemeral and therefore can survive light exposure if at all only for a short time. Their ascomata develop in shaded or permanently dark ecosystems such as karstic pits and caves (*R. ozimecii*) [64], where UV radiation could be less problematic during the development of the ascospores.

### 4.4. Evolution in Thelebolales Lineages

Based on morphology and ecology, it is initially surprising that *Holwaya* and *Patinella* (*Holwayaceae*) are related to *Thelebolaceae*-*Pseudeurotiaceae* lineages; however, the phylogenetic evidence in our multigene analyses is clear (Figure 1) and confirms previous results [14]. There are several interesting examples of homoplasy among *Holwayaceae* and genera in *Pseudeurotiaceae* and *Thelebolaceae*. *Holwaya* and *Patinella* species have asci with OLR and paraphyses with enlarged apices, something that evolved independently in some *Thelebolus* species with active ascospore discharge (Figure 2, Figure 3, Figure 5 and Figure 8). However, the most striking similarity is between *T. microsporus* (*Thelebolaceae*) and the oldest lineage in *Holwayaceae*, the genus *Ramgea*. Both share the following traits [60,64,90,106]: (1) paragymnohymenial ontogeny and closed ascoma in early development that opens late upon ascospore maturation (Figure 2, Figure 6 and Figure 7); (2) thin, lateral, simply structured excipular flanks composed of ± isodiametric, slightly yellowish-grey cell walls (Figure 5, Figure 6 and Figure 7); (3) abruptly enlarged apices of the paraphyses with similar pigmentation inside the cells (Figure 5 and Figure 6); (4) the presence of a wall reinforcement in the asci that limits the area of the apical opening (OLR) (Figure 6 and Figure 8); (5) thick-walled, hyaline, aseptate ascospores (Figure 3, Figure 4, Figure 5, Figure 6 and Figure 7); (6) the presence of gluing substances or TEM microtubules among the ascospores to facilitate the active release of all spores as a single unit [107]; and (7) ecology (dung saprobes). Due to these similarities, in the past, these genera were placed together within the same family (*Thelebolaceae*) [65]. However, this convergent evolution is likely a response to similar lifestyles [108]: habitat (cold environments), substrate (dung), ecology (saprotrophs), and a similar mechanism for dispersal (asci with OLR, Figure 8). The ascospores of these dung inhabitors need to actively escape their habitat, “sinking islands”, an ephemeral source of nutrients, and reach a distance to avoid the zone of repugnance and be ingested/transported by animals [16,60,99,109]. Due to OLR asci and the presence of adhering ascospores [16,64,106,107], ascospores are ejected as a single projectile, which helps increase shooting distance, a common phenomenon in coprophilous fungi [16,19,21,26,32]. In this case, the thick wall of the ascospores protects against hazards of gut passage (pH, digestive enzymes, etc.) [60,110,111].

The ancestor of *Thelebolaceae*/*Pseudeurotiaceae* was reconstructed to have had a closed ascoma, without paraphyses, and with evanescent asci that contain ellipsoid-fusoid ascospores (Figure 2, Figure 3, Figure 5 and Figure 7). The *Thelebolaceae* clade has achieved adaptive radiation in all traits analyzed compared with all other lineages, where a smaller number of changes through their evolution were found. Only the shape and color of ascospores were constant in *Thelebolaceae*. Species in the family evolved different types of ascomata (open, closed, or naked), asci (active or passive discharge), the presence or absence of paraphyses, and changes in ascospore number and ornamentation (Figure 2, Figure 3, Figure 4, Figure 5 and Figure 7). *Cleistothelebolus* retains all the ancestral traits of *Thelebolales*, as does *Pseudeurotium* (Figure 2, Figure 3, Figure 4 and Figure 5, clade N and J). Species of both genera have closed ascomata that are considered cleistothecia (Figure 7), with a membranaceous peridium composed of polygonal cells, and the ascogenous system spread irregularly throughout the ascomatal interior [112]. In other closed ascomatal lineages, such as *Bettsia*, *Gymnostellatospora*, *Leuconeurospora*, and *Pseudogymnoascus* (*Pseudeurotiaceae* s.l.), the peridium evolved differently, although they also have a similar ascogenous arrangement [17]. Conversely, species with closed ascoma in *Thelebolus* have an ascogenous system regularly arranged in a hymenial layer [60]. The loss of active ascospore discharge in *Cleistothelebolus* and *Pseudeurotium* likely afforded more effective protection during ascosporogenesis, opening only after the ascospores are completely mature and capable of withstanding environmental stresses [112,113]. *Cleistothelebolus* is sister to *Thelebolus*, and both genera have similar ecologies (mostly isolated from dung), and its morphology is similar to those species of *Thelebolus* with closed ascoma (Figure 2, Figure 3, Figure 4, Figure 5, Figure 6 and Figure 7). However, *Cleistothelebolus* has a narrow optimum temperature for fruiting of around 25 °C and evolved in adaptation to warmer environments compared to the more psychrotolerant/psychrophilic *Thelebolus* [60,112]. Although they are not closely related, *Cleistothelebolus* and *Pseudeurotium* share all traits except ascospore color (hyaline vs. dark) and the composition of the cover layer of the ascoma (i.e., arrangement of cells), which is one layer in *Cleistothelebolus* and several in *Pseudeurotium* (Figure 4, Figure 5 and Figure 7). They also dwell in different habitats. *Cleistothelebolus* has been only recorded a few times from carnivore dung (*op. cit.*), but *Pseudeurotium* is a cosmopolitan genus that has been reported as an endophyte in algae, isolated from grass, wood, freshwater, marine sponges, soil (including permafrost), diesel fuel, dung, and sea-bottom sediments [113,114,115,116,117,118]. Despite these many reports, the ecology of *Pseudeurotium*, as well as its dispersal mechanism, remains unclear. *Pseudeurotium* species have evolved in adaptation to harsh habitats (heat, cold, osmotic pressures, etc.), where whole ascomata and ascospores are likely dispersed by wind/water. Hoog et al. [60] suggested water dispersal for species of *Thelebolus* with similar traits. Species of *Pseudeurotium* have two interesting apomorphies: small globose ascospores that are dark-walled (Figure 3, Figure 4, Figure 5, Figure 6 and Figure 7). These spore traits are found in species that evolved long-distance dispersal [29,30,37,38,40,41,44,45,47].

*Thelebolus* species are psychrophilic and have been isolated from dung, mud, soil, fresh or saltwater, the digestive tracts of birds, marine sponges, and living plant tissue such as endophytes. Ascospore production is enhanced by temperatures below 15 °C [60,106]. Our analyses inferred the ancestor of *Thelebolus* as having a closed ascoma, without paraphyses, and with passive ascospore dispersal (Figure 2, Figure 3, Figure 4 and Figure 5, clade O). Most species of *Thelebolus* have these traits [60,106,119], although there are some species with open ascoma that actively discharge ascospores. Hoog et al. [60] postulated that species with open ascomata and active ascospore discharge have an ancestral position in the genus due to their broad distribution and correlation with animal dispersal (birds, mammals). They considered that species with closed ascomata (*T. ellipsoideus*, *T. globosus*) evolved in response to cold environments and the loss of bird vectors. Our results and evolutionary hypothesis regarding *Thelebolus* disagree with Hoog et al. [60]. We found that the closed ascoma is an ancestral trait in *Thelebolaceae* (Figure 2 and Figure 5 clade M) and in *Thelebolus* (Figure 2 and Figure 5, clade O). Therefore, the reappearance of apothecial ascomatal configuration with forcible spore discharge in some *Thelebolus* species (Figure 2, Figure 5 and Figure 7) is an apomorphy. When complex phenotypic traits are lost in evolution, evolutionary reversals are unlikely (i.e., Dollo’s law of irreversibility); for example, the loss of the flagellum was one of the major changes in fungal evolution, a trait that has not been gained again after its loss [56]. However, examples of evolutionary reversals are known, e.g., in *Animalia*: [120,121,122,123]. The loss of active ascospore discharge is correlated with closed ascoma development and has evolved multiple times in several lineages of *Leotiomycetes* (*Amorphothecaceae*, *Helotiaceae*, *Myxotrichaceae*, *Pseudeurotiaceae*, *Rutstroemiaceae*, *Thelebolaceae*), as well as in other lineages of lichenized and non-lichenized *Ascomycota* [11,14,25,54]. Regaining a trait represents a rare reversal phenomenon that could be designated as a “re-evolution” [121,122,123,124]. We have been unable to find any example in *Leotiomycetes* that represents a re-evolution. Therefore, it is likely that some *Thelebolus* species could provide the first known example of re-evolution in this class (Figure 2, Figure 3, Figure 4 and Figure 5, Figure 7 and Figure 8).

Our analyses showed another homoplastic trait, ornamented ascospores, which evolved multiple times in different lineages: *Holwayaceae* (*Ramgea*), *Pseudeurotiaceae* (*Gymnostellatospora*, *Leuconeurospora*, *Pseudogymnoascus*), and *Thelebolaceae* (*Antarctomyces*) (Figure 4, Figure 5, Figure 6 and Figure 7). Ornamented ascospores are not common in *Leotiomycetes* [25]. Ornaments of spores are generally hydrophobic and tend to facilitate attachment to substrates [125,126]. Ornamented ascospores are present in many diverse, animal-associated fungi with enclosed or partially enclosed ascomata, i.e.,: *Onygenales* (*Arthrodermataceae*, *Gymnoascaceae*, *Myxotrichaceae*, *Onygenaceae*), *Pezizales* (*Tuberaceae*), *Cephalothecales* (*Cephalothecaceae*), and *Eurotiales* (*Elaphomycetaceae*) [15,18,103]. We can observe a similar evolution pattern in some lineages of *Pseudeurotiaceae* (Figure 4 and Figure 5 clade D). Vanderwolf et al. [127] found that arthropods contribute to the dispersal of *Pseudogymnoascus* and *Leuconeurospora*; species of both genera have been also isolated from the fur and skin of hibernating bats [128]. *Leuconeurospora* has closed ascomata with a peridial cover modified into groups of radiating cells with well-defined lines of dehiscence (Figure 7). This special peridial covering is called cephalothecoid and has been correlated with arthropod dispersal [103]. *Pseudogymnoascus* and *Gymnostellatospora* have cage- or mesh-like closed ascomata (Figure 7) that are very similar to the reticuloperidial gymnothecia in *Myxotrichaceae*. These highly reduced ascomata have also evolved to be dispersed by insects [15]. An example of extreme simplification in *Thelebolales* adapted to zoochory is the beehive-dwelling *Bettsia*. This genus, and others that share the same phenotypic traits have a predilection for high-sugar substrates (osmophilic) where water activity is low and solute concentration is high [17,129]. Wynns [17] showed the convergent evolution between *Ascosphaera* (*Eurotiomycetes*) vs. *Bettsia* (*Thelebolales*, *Leotiomycetes*) and *Eremascus* (*Eurotiomycetes*) vs. *Skoua* (*Myxotrichiaceae*, *Leotiomycetes*). These taxa evolved independently to produce highly simplified closed ascomata. In *Ascosphaera* and *Bettsia*, the closed ascoma is a cyst-like cell envelope with evanescent asci instead of a more complex multicellular peridium (Figure 7). A further reduction is found in a species of *Eremascus* and *Skoua*, in which ascomata are absent and asci are produced directly on hyphae, such as in *Antarctomyces* (Figure 2, Figure 5 and Figure 7). Wynns [17] suggested that cyst-like and naked asci are reduced forms of cage- or mesh-like closed ascomata (i.e., reticuloperidial ascomata). Our results showed *Bettsia* as a basal lineage of *Pseudeurotiaceae*/*Thelebolaceae* and *Antarctomyces* as a basal lineage of *Thelebolaceae*. Therefore, we have not found the evolution pattern for *Bettsia* and *Antarctomyces* (Figure 2 and Figure 5 clade B2, L) suggested by Wynns [17] for *Ascosphaera* and *Eremascus*. We believe that more sequence data and taxon sampling from those genera currently without sequences in *Thelebolales* are needed to determine the real affinities of these two genera inside the order.

### 4.5. Known Anamorphs and Conidia Dispersal

Anamorphs in *Holwayaceae* are known only for *Holwaya*. The anamorph of *H. mucida* is conspicuous and readily identifiable in the field, where it occurs scattered gregariously and sometimes covering large areas (up to several meters long) of host logs [89] (Matočec and Kušan pers. obs.). Its determinate synnemata are 2 mm in diameter and up to 11 mm tall, with shiny black stipes and grey fertile heads comprised of branched, hyaline conidiophores with phialides producing aseptate, ellipsoidal, hyaline, smooth conidia, in a slimy mass. The conidia frequently germinate by budding to form microconidia [87,89,130]. The dispersal mode of *Holwaya* conidia is unknown, but its slimy conidia are likely dispersed by insects, rain splash, and surface water films [131,132]. Furthermore, the ability of *Holwaya* species to inhabit logs with reduced or fluctuating water availability may be enhanced by this conidial phase, which enables it to produce propagules in less favorable ecological conditions when apothecial development is suppressed [133,134]. The synnemata of *Holwaya* are distinct from all other known anamorphs in *Thelebolales*. *Thelebolaceae* anamorphs are morphologically reduced, with *Thelebolus ellipsoideus* and *T. globosus* forming yeast-like morphs involving integrated, intercalary conidiogenous cells producing aseptate, subglobose or ellipsoid, hyaline, thin-walled, smooth conidia [60]. Similarly, *Inopinatum lactosum* colonies are dimorphic and yeast-like, producing blastoconidia [59]. *Cleistothelebolus nipigonensis* also forms a yeast-like anamorph, with integrated, intercalary, or peg-like conidiogenouscells producing aseptate, ellipsoidal to short cylindrical, hyaline, thin-walled, smooth conidia in basipetal order from protruding scars; the conidia adhere to the conidiogenous cells in slimy globules and often turn into budding cells that produce daughter cells [112,135]. *Antarctomyces psychotrophicus* forms a sporothrix-like morph consisting of enteroblastic, integrated conidiogenous cells producing aseptate, subglobose to irregularly cylindrical, hyaline, thick-walled, smooth conidia that aggregate in slimy masses [136]. *Antarctomyces pellizariae* forms an anamorph similar to *A. psychotrophicus* and both species also produce abundant irregular, 1–2-celled chlamydospores singly or in long chains [137]. The formation of yeast-like colonies is apparently rare in *Leotiomycetes* [138], but examples exist in *Thelebolaceae*, as listed above. The production of yeast-like, budding conidia in slimy masses may be an adaptation for dispersal to new sources of dung via coprophilous arthropods. For example, some strains of *Cleistothelebolus nipigonensis* produce wine- or cheese-like odors that might function as an insect attractant [135,139]. In *Thelebolus* spp. isolated from birdless lakes covered by ice for all or most of the year, Hoog et al. [60] suggested that the loss of bird vectors led to an increasing predominance of conidial production, and a reduction in asci or the absence of sexual reproduction. The authors also suggested that *T. globosus* thrived in submerged conditions for a major part of its life cycle, evinced by its loss of active ascospore discharge and abundantly produced simple, slimy conidia that are water dispersed.

*Geomyces* (*Pseudeurotiaceae*) anamorphs typically consist of aseptate, globose, barrel-shaped, pyriform, or clavate conidia that are hyaline (sometimes yellow), thin-walled, smooth, or echinulate. The conidia are borne apically (aleurioconidia) and often intergraded with intercalary conidia produced from short, distinct, branching (sometimes verticillately) conidiophores. *Geomyces*-like anamorphs are also found in *Pseudogymnoascus* and *Solomyces* [86,140]. *Pseudeurotium* anamorphs are sporothrix-like, with conidiophores consisting of a single terminal or intercalary conidiogenous cells with short side branches bearing aseptate, globose, obovoid to ellipsoidal, hyaline conidia (sometimes turning dark brown after prolonged storage) borne singly or in clusters from short denticles at the apices [141]. Reduced malbranchea-like alternate arthroconidia are reported for *Gymnostellatospora* [142]. *Leuconeurospora* species may form both arthroconidia and dichotomously branched conidiophores with annellides that produce chains of aseptate, obovate to pyriform, basally truncate, thin- or thick-walled, hyaline to dark green, smooth conidia [128,143,144]. *Bettsia alvei* and *B. fastidia* (*Pseudeurotiaceae*) anamorphs were previously described in *Chrysosporium* and consist of solitary aleurioconidia borne on short pedicels and intercalary chlamydospores or arthroconidia [129]. Animals (arthropods, birds, mammals including humans) and air currents appear to be important dispersal agents of *Pseudeurotiaceae* conidia [128,145,146,147]. The majority of reticuloperidial fungi (including *Pseudeurotiaceae*) produce dry, cylindrical arthroconidia, which may enhance their electrostatic attraction to arthropods compared to spherical conidia with lower surface area [15].

Although the complex synnemata of *Holwaya* distinguish *Holwayaceae* from all other *Thelebolales* taxa, culture studies are needed to better understand the morphological diversity of the anamorphs of *Holwayaceae* and *Thelebolales*. The frequent isolation of *Thelebolales* species from environmental samples is suggestive of psychrotolerance, halotolerance, xerotolerance, dimorphism, and temperature-controlled sporulation [148]. Culture studies should include different salt, osmolyte, and temperature treatments to attempt to induce sporulation and provide physiological data that may in turn infer ecological traits.

## 5. Conclusions

Apothecial ancestry and evolution patterns in *Thelebolales* are revealed by the inclusion of the new family *Holwayaceae*. *Holwayaceae* contains three genera (*Holwaya*, *Patinella*, *Ramgea*) with wide morphological and ecological diversity, for example, the apothecial and conidial morphologies of the wood saprobe *Holwaya* diverge from other *Thelebolales* taxa. In *Thelebolales*, evanescent asci are surrounded by an extraordinary variety of peridial walls from different types of closed ascomata (cleistothecial, gymnothecial). Species with closed ascomata protect ascospores, which are passively discharged as an adaptation to similar ecologies (soil, dung saprobes), harsh macroclimate conditions (cold, hot), and microenvironments (osmotic pressures). The water and wind dispersal of ascospores is suggested in some lineages, but clear examples of phenotypic adaptation to animal dispersal are shown in *Pseudeurotiaceae* and *Thelebolaceae*. In response to the absence of animal (bird) vectors, *Thelebolus* spp. are believed to produce cleistothecial ascomata with evanescent asci and to rely increasingly on asexual reproduction. Yeast-like conidial morphs are relatively rare in *Leotiomycetes*, but several examples can be found in *Thelebolaceae* and *Pseudeurotiaceae*, which might be an adaption to dispersal by invertebrates and/or a stress response to cold and marine habitats. Many *Thelebolales* species occupy extreme habitats and exhibit corresponding adaptations. Highly reduced ascomata, from cyst-like (in adaptation to low water-activity substrates in *Bettsia*) to naked asci in cold environments (*Antarctomyces*) evolved independently and are not derived from cage- or mesh-like closed ascomata in *Thelebolales*. *Holwayaceae*, the basal family of the order, retained all phenotypic traits of the ancestor of *Thelebolales*, namely apothecioid, paraphysate ascomata with actively discharging asci. Phenotypic variation in ascoma size, cell pigmentation, ascus, and ascospore shape and biometry are correlated with nutritional strategies in this family. Furthermore, the dung saprobes *Ramgea* (*Holwayaceae*) and *Thelebolus* (*Thelebolaceae*) evolved independently but share multiple traits, including the homoplasic mechanism of ascus opening (OLR) and actively discharging asci, which, in *Thelebolus*, represents the first report of the phenomenon of re-evolution in *Leotiomycetes*.

## Figures and Tables

**Table 1:** A list of species, collection numbers, GenBank accession numbers used in this study, and phenotypic features with character states given for each species representative. N/A: not available. Asc = ascoma type; Dis = ascospore discharge; Par = paraphyses; SpMor = ascospore morphology; SpCol = ascospore color; SpOrn = ascospore ornamentation.

Species	Collection Number	*ITS*	*LSU*	Asc	Dis	Par	SpMor	Sp	Sp
								Col	Orn
*Antarctomyces pellizariae*	UFMGCB 12416	KX576510	N/A	2	1	1	1	0	1
*Antarctomyces psychrotrophicus*	VKM-4686	MF375780	MF375780	2	1	1	1	0	1
*Aotearoamyces nothofagi*	PDD:55517	KM677201	MG807387	0	0	0	0	0	0
*Bettsia alvei*	CBS 487.91	MH862266	MH873948	1	1	1	2	1	0
*Claussenomyces prasinulus*	CBS 111551	KX090866	KX090815	0	0	0	0	0	0
*Cleistothelebolus nipigonensis*	CBS 778.70	KC492060	MH871738	1	1	1	1	0	0
*Gymnostellatospora alpina*	CBS 620.81	MH861383	MH873132	1	1	1	1	0	1
*Gymnostellatospora bhattii*	CBS 760.71	MH860337	MH872092	1	1	1	1	0	0
*Gymnostellatospora canadensis*	UAMH 8899	NR111199	N/A	1	1	1	1	0	1
*Gymnostellatospora japonica*	VKM-4687	MF375781	MF375781	1	1	1	1	0	1
*Holwaya mucida*	CNF 2/8749	OM282975	OM282978	0	0	0	0	0	0
*Leotia lubrica*	KKM 337	KF836617	KF836627	0	0	0	0	0	0
*Leuconeurospora polypaeciloides*	UAMH 11459	KC884266	N/A	1	1	1	1	0	1
*Leuconeurospora pulcherrima*	CBS 343.76	KF049206	FJ176884	1	1	1	1	0	1
*Microglossum olivaceum*	FH-DSH97-103	AY789398	AY789397	0	0	0	0	0	0
*Myriodiscus sparassoides*	KKUK2	JX219380	JX219382	0	0	0	2	0	0
*Patinella hyalophaea*	H.B. 9739	KT876978	KT876978	0	0	0	1	0	0
*Pseudeurotium bakeri*	CBS 878.71	NR145345	N/A	1	1	1	2	1	0
*Pseudeurotium desertorum*	CBS 986.72	JX076946	N/A	1	1	1	2	1	0
*Pseudeurotium ovale*	FMR 13600	KP686192	KP686193	1	1	1	1	1	0
*Pseudeurotium zonatum*	CBS 329.36	NR111127	DQ470988	1	1	1	2	1	0
*Pseudogymnoascus appendiculatus*	02NH11	JX270356	KF017819	1	1	1	1	0	0
*Pseudogymnoascus lindneri*	02NH05	JX270350	KF017818	1	1	1	1	0	0
*Pseudogymnoascus roseus*	05NY06	JX270385	KF017824	1	1	1	1	0	0
*Pseudogymnoascus turneri*	CUP-070715	MN542214	N/A	1	1	1	1	0	0
*Pseudogymnoascus verrucosus*	04NY16	JX270377	KF017822	1	1	1	1	0	1
*Ramgea ozimecii*	CNF 2/9997	KY368752	KY368753	0	0	0	1	0	1
*Thelebolus balaustiformis*	MUT 2357	NR159056	MG816492	0	0	1	1	0	0
*Thelebolus ellipsoideus*	CBS 113937	AY957550	FJ176895	1	1	1	1	0	0
*Thelebolus globosus*	AFTOL-ID 5016	KM822751	FJ176905	1	1	1	1	0	0
*Thelebolus microcarpus*	CBS 137501	LN609269	MH877647	1	1	1	1	0	0
*Thelebolus microsporus*	CBS 115.53	16145654	16145752	0	0	0	1	0	0
*Thelebolus spongiae*	MUT 2359	MG813185	MG816493	1	1	1	1	0	0
*Thelebolus stercoreus*	CBS 717.69	MH859395	MH871166	0	0	1	1	0	0
*Thuemenidium atropurpureum*	ILLS:61044	JQ256427	JQ256441	0	0	0	0	0	0
*Tympanis tsugae*	LQH-8	MH810146	MH810148	0	0	0	2	0	0

## Data Availability

The data presented in this study are openly available in Manaaki Whenua–Landcare Research Datastore at https://doi.org/10.7931/X93K-H703. Publicly available datasets were also analyzed in this study. This data can be found here: www.ncbi.nlm.nih.gov/genbank/ (accessed on 1 July 2021).

## Supplementary Materials

The following supporting information can be downloaded at: https://doi.org/10.7931/X93K-H703.
